# Short-term effect of anti-VEGF for chronic central serous chorioretinopathy according to the presence of choroidal neovascularization using optical coherence tomography angiography

**DOI:** 10.1371/journal.pone.0245342

**Published:** 2021-01-11

**Authors:** Yong-Yeon Song, Hwa-Young Yu, Seung-Kook Baek, Young-Hoon Lee, Min-Woo Lee

**Affiliations:** Department of Ophthalmology, Konyang University College of Medicine, Daejeon, Republic of Korea; Icahn School of Medicine at Mount Sinai, UNITED STATES

## Abstract

**Purpose:**

To analyze the short-term therapeutic efficacy of intravitreal injection of bevacizumab (IVB) for chronic central serous chorioretinopathy (CSC) according to the presence of choroidal neovascularization (CNV) using optical coherence tomography angiography (OCTA).

**Methods:**

A retrospective chart review was perfomed on cases of CSC with CNV (Group 1: n = 31) and an age-matched cases of CSC without CNV (Group 2: n = 30). The response to IVB was evaluated by changes in best-corrected visual acuity (BCVA), central macular thickness (CMT), choroidal thickness (CT), and pachyvessel diameter. Univariate and multivariate linear regression analyses were performed to identify factors associated with the visual outcome of chronic CSC with CNV after IVB.

**Results:**

At baseline, the CT values differed significantly between Groups 1 and 2 (371.55 ± 67.09 vs. 417.33 ± 71.32 *μ*m, p = 0.01). In Group 1, BCVA improved significantly (p < 0.001), and CMT (p < 0.001), CT (p = 0.001) and pachyvessel diameter (p = 0.045) decreased significantly, after IVB. In Group 2, only pachyvessel diameter (p = 0.001) was significantly smaller after IVB. Univariate analysis showed that the initial CT (B = 0.002, p = 0.026) and pachyvessel diameter (B = 0.002, p = 0.001) significantly affected visual outcome. In multivariate analysis, the initial pachyvessel diameter exhibited significant results (B = 0.002, p = 0.001).

**Conclusions:**

IVB showed less effective short-term outcomes in chronic CSC patients without CNV than in patients with CNV. In chronic CSC with CNV, the short-term visual outcome after IVB was better in patients with a thinner choroid and smaller pachyvessels.

## Introduction

Central serous chorioretinopathy (CSC) is characterized by serous retinal detachment with or without retinal pigment epithelial (RPE) detachment, which usually involves the macula.[1] The precise pathogenesis is not known, but may be associated with choroidal hyperpermeability as a result of stasis, ischemia, and inflammation [2]. CSC usually has a good prognosis characterized by spontaneous regression of subretinal fluid (SRF) and symptoms within 4 months; only 5–10% of patients experience chronic or recurrent CSC which results in RPE atrophy and severe vision loss [3]. Chronic CSC is characterized by persistent SRF and widespread RPE alterations, including photoreceptor elongation, subretinal fibrosis and, occasionally choroidal neovascularization (CNV) [4,5]. CNV develops in 4–8% of chronic CSC patients [6,7]. There are various treatment options such as photodynamic therapy (PDT), focal laser photocoagulation, and anti-vascular endothelial growth factor (VEGF) intravitreal injection. Several studies reported that anti-VEGF treatment was effective for chronic CSC patients [8–10]. Anti-VEGF agents also have an important role in treating chronic CSC with CNV [11].

Optical coherence tomography angiography (OCTA) is a non-invasive imaging modality that has enabled direct visualization of the retinal circulation, in a multilayered, three-dimensional way [12,13]. This technique, which does not involve injection of dye, is effective for defining the shape of the neovascularization (NV) and clearly distinguishing it from the surrounding tissue [14]. For chronic CSC, the presence of CNV has been reported as a major risk factor for a poor visual outcome [15,16]. Several studies have used OCTA to determine the presence of CNV in CSC, with variable results [17–19]. Bousquet et al. [20] found that, in patients with chronic CSC, 35.6% of eyes with areas of flat irregular pigment epithelial detachment showed CNV. Using OCTA, Savastano et al. [21] retported that the incidence of CNV in CSC was 20%, which was higher than that of previous studies. In both studies, it was emphasized that OCTA can better reveal the presence of NV than other imaging modalities. However, few studies have reported the outcome of anti-VEGF treatment for chronic CSC according to the presence or abscence of CNV using OCTA.

The purpose of this study was to evaluate the short-term therapeutic efficacy of intravitreal injection of bevacizumab (IVB) for chronic CSC according to the presence of CNV using OCTA. Additionally, we evaluated factors associated with the visual outcome of chronic CSC with CNV treated by IVB.

## Methods

### Patients

This study adhered to the tenets of the Declaration of Helsinki and was approved by the Institutional Review Board of Konyang University Hospital, Republic of Korea (2020-09-003). Informed consent was waived due to the retrospective nature of the study. This case-control study of short-term effect of IVB (1.25 mg/0.05 mL dosage) on chronic CSC patients with and without CNV was conducted at our clinic from May 2017 to August 2019. Chronic CSC is defined as the presence of visual symptoms for at least 6 months with clinical features of CSC, including SRF and RPE changes in the macular region on spectral-domain optical coherence tomography (SD-OCT; Heidelberg Engineering, Heidelberg, Germany), active angiographic leakage in fluorescein angiography (FA; Heidelberg Engineering, Heidelberg, Germany) and abnormal dilated choroidal vasculature with hyperpermeability in indocyanine green angiography (ICGA; Heidelberg Engineering, Heidelberg, Germany). All patients underwent a routine ophthalmic examination, including best-corrected visual acuity (BCVA), intraocular pressure (IOP), spherical equivalent (SE), and axial length using the IOL Master (Carl Zeiss, Jena, Germany). We excluded patients with a history of previous treatment, such as PDT or IVB which could have affected the result. The second exclusion criterion was any retinal diseases other than CSC. Color and autofluorescence fundus photographs were analyzed to identify any sign of age-related macular degeneration (AMD), such as macular drusen.

### OCT and OCTA measurements

**Fig 1:**
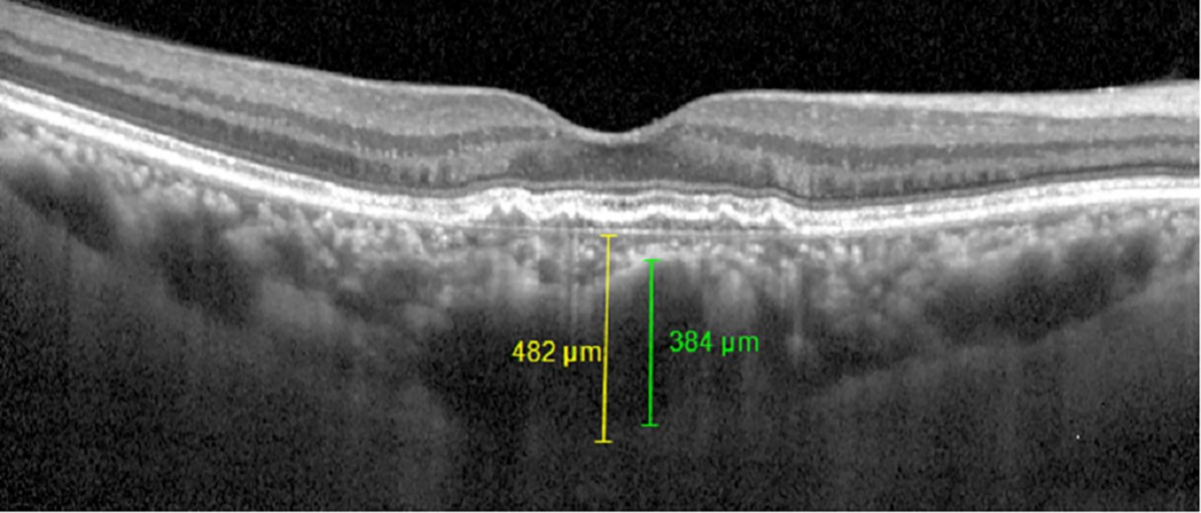
Choroidal thickness and pachyvessel diameter measurement on spectral domain (SD) optical coherence tomography with enhanced depth imaging (EDI) mode. Choroidal thickness (yellow line) was obtained by measuring the perpendicular distance from the outer layer of the RPE to the inner surface of the sclera at the subfoveal area using software calipers. Pachyvessel diameter (green line) was obtained by measuring the vertical diameter of the largest outer choroidal vessel in the foveal area using software calipers.

The patients were divided into two groups according to the presence of CNV as determined by OCTA measurement. After enrollment of CSC with CNV patients (group 1), CSC without CNV patients (group 2) were randomly matched by age to the group 1. OCTA and SD-OCT were performed using the Spectralis OCT2 instrument, which is capable of 70,000 A-scans/s using a light source centered at 870 nm, and with axial and transverse resolutions of 3.9 and 6 μm in tissue, respectively. En face OCT-A images were recorded with a 20° × 15° angle and lateral resolution of 5.7 μm/pixel, resulting in a retinal section of 2.9 mm × 2.9 mm. We obtained the data of central macular thickness (CMT), choroidal thickness (CT), pachyvessel diameter, choroidal caverns at the times of before and 1 month after IVB using SD-OCT with enhanced depth imaging mode (Fig 1). CMT is defined as the average macular thickness in the central 1 mm Early Treatment Diabetic Retinopathy Study grid, and was measured automatically using bundled software. The CT was obtained as an average value, by measuring the perpendicular distance from the outer layer of the RPE to the inner surface of the sclera using software calipers at the subfoveal area. The pachyvessel diameter is defined as the vertical diameter of the largest outer choroidal vessel in the foveal area, and was also measured using software calipers as previous studies [22–24]. Choroidal caverns are identified as focal hyporeflective spaces on B-scan images and its numbers are counted. CT, pachyvessel diameter and choroidal caverns were measured manually by two graders (YYS and HYY) who were blinded to each other’s measurements.

### Statistical analyses

Statistical analysis was performed using PASW Statistics (ver. 20; SPSS Inc., Chicago, IL, USA); values are expressed as the mean ± standard deviation. Categorical variables were compared between the two groups using the chi-square test. Continuous variables were analyzed using Student’s t-test, and paired data using a paired t-test. In the chronic CSC with CNV group, univariate and multivariate generalized linear regression were used to identify factors affecting visual acuity after treatment. A p-value < 0.05 was considered statistically significant.

## Results

### Demographics

We reviewed 31 eyes of CSC with CNV (group 1) and 30 eyes with CSC without CNV (group 2). The baseline characteristics of the patients before treatment are shown in Table 1.

**Table 1:** Baseline characteristics of Group 1 (CSC with CNV), and Group 2 (CSC without CNV).

Variables	Group 1 (w/CNV) (n = 31)	Group 2 (w/o CNV) (n = 30)	p-value
Age (years)	51.42 ± 10.76	50.93 ± 9.53	0.85
Sex (M: F)	24: 7	27: 3	0.19
Laterality (R: L)	15: 16	10: 20	0.23
IOP (mmHg)	13.47 ± 2.92	12.8 ± 2.82	0.37
Spherical equivalent	−0.41 ± 1.76	−0.22 ± 1.15	0.31
BCVA	0.31 ± 0.34	0.23 ± 3.0	0.37
CMT (*μ*m)	350.65 ± 74.01	322.73 ± 90.23	0.19
CT (*μ*m)	371.55 ± 67.09	417.33 ± 71.32	**0.01**
Pachyvessel diameter (*μ*m)	235.48 ± 76.12	249.80 ± 71.12	0.45
Choroidal cavern	0.81 ±1.64	0.7 ± 0.92	0.76

IOP, Intraocular pressure; BCVA, best-corrected visual acuity; CMT, central macular thickness; CT, choroidal thickness.

p-values <0.05 that are statistically significant are in bold font.

The mean age was 51.42 ± 10.76 and 50.93 ± 9.53 years, the mean SE was −0.41 ± 1.76 and −0.22 ± 1.15 diopters, and the BCVA was 0.31 ± 0.34 and 0.23 ± 3.0 for groups 1 and 2, respectively. The respective CT values were 371.55 ± 67.09 and 417.33 ± 71.32 *μ*m; the difference was significant (p = 0.01). The respective CMT values were 350.65 ± 74.01 and 322.73 ± 90.23 *μ*m; those for pachy vessels was 235.48 ± 76.12 and 249.80 ± 71.12 *μ*m; and the numbers of choroidal cavern was 0.81 ± 1.64 and 0.7 ± 0.92, respectively. The values measured manually by the two graders exhibited excellent interobserver reproducibility (ICC > 0.95, CV < 5%).

### Visual outcome and OCT parameters after treatment in both groups

In Group 1, BCVA improved significantly at 1 month of IVB from 0.31 ± 0.34 to 0.24 ± 0.32 (p < 0.001) (Table 2).

**Table 2:** Visual and anatomical outcomes of both groups before and 1 month after treatment.

	Group 1 (w/CNV)			Group 2 (w/o CNV)		
	Before	After	p-value	Before	After	p-value
BCVA	0.31 ± 0.34	0.24 ± 0.32	**<0.001**	0.23 ± 3.0	0.26 ±0.39	0.432
CMT (*μ*m)	350.65 ± 74.01	276.42 ± 58.34	**<0.001**	322.73 ± 90.23	305.67 ± 113.9	0.301
CT (*μ*m)	371.55 ± 67.09	347.1 ± 64.71	**0.001**	417.33 ± 71.32	413.67 ± 66.12	0.227
Pachyvessel diameter (*μ*m)	235.48 ± 76.12	224 ± 72.23	**0.045**	249.80 ± 71.12	234.37 ± 77.08	**0.001**
Choroidal Cavern	0.81 ±1.64	0.74 ± 1.53	0.625	0.7 ± 0.92	0.67 ± 0.98	0.861

BCVA, best-corrected visual acuity; CMT, central macular thickness; CT, choroidal thickness.

p-values < 0.05 that are statistically significant are in bold font.

**Fig 2:**
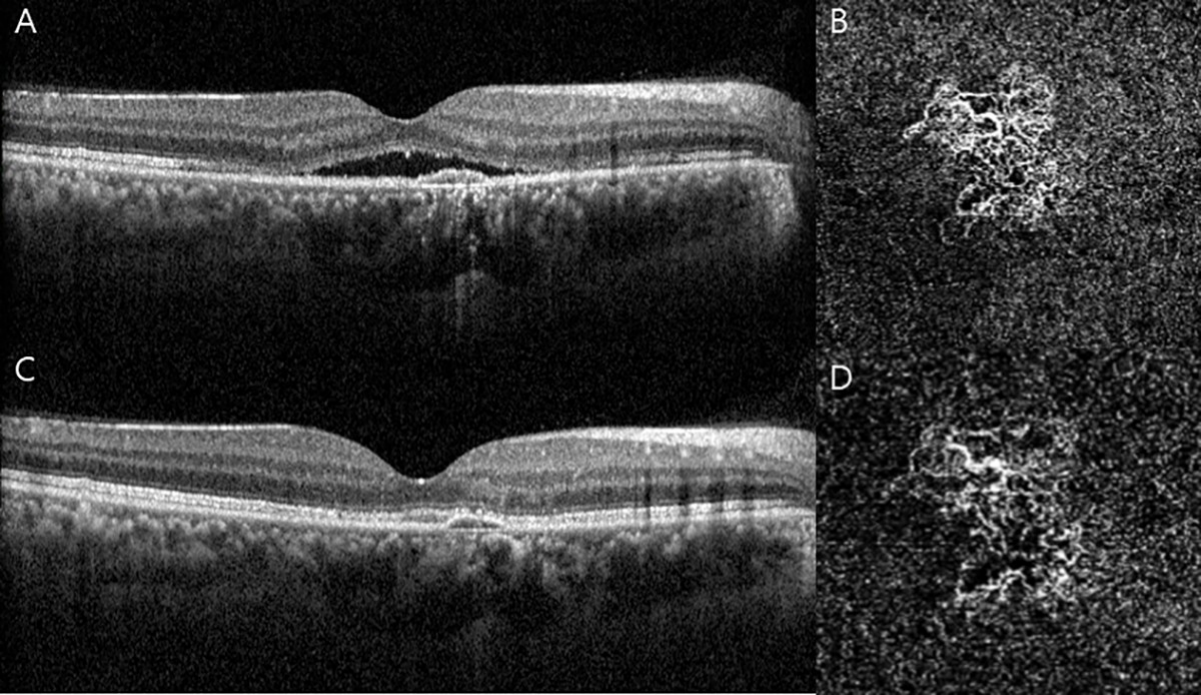
Representative images of chronic central serous chorioretinopathy (CSC) with choroidal neovascularization (CNV) (Group 1). The upper row: before treatment (A) Horizontal B-scan of optical corerence tomography (OCT) centered to the fovea showing subretinal fluid (SRF) with pigment epithelial detachment (PED). (B) Enface OCT angiography (2.9 x 2.9mm) through the choriocapillaris shows CNV below the PED. The lower row: 1 month after intravitreal injection of bevacizumab (IVB). (C) Shows complete resolution of SRF. (D) Shows a smaller area of the CNV with reduction of the dense microvasculature.

CMT were significantly reduced after IVB treatment from 350.65 ± 74.01 to 276.42 ± 58.34 *μ*m (p < 0.001) (Fig 2).

CT were also significantly reduced after IVB treatment from 371.55 ± 67.09 to 347.1 ± 64.71 *μ*m (p = 0.001). Pachyvessel diameter were significantly decreased after IVB treatment from 235.48 ±76.12 to 224 ± 72.23 *μ*m (p = 0.045). In Group 2, only pachyvessel diameter showed significant decrease after 1 month of IVB treatment from 249.80 ± 71.12 to 234.37 ± 77.08 *μ*m (p = 0.001), and no significant difference in choroidal cavern numbers was observed in either groups. We also measured differential values of BCVA, CMT, CT, pachyvessel diameter, the numbers of choroidal cavern between before and 1 month after IVB, between Groups 1 and 2 (Table 3).

**Table 3:** Effects of intravitreal bevacizumab injection in both groups.

Variables	Group 1 (w/CNV) (n = 31)	Group 2 (w/o CNV) (n = 30)	P-value
BCVA change	0.07 ± 0.09	−0.03 ± 0.19	**0.012**
CMT change (*μ*m)	74.23 ± 60.52	17.07 ± 88.80	**0.005**
CT change (*μ*m)	24.45 ± 36.88	3.67 ± 16.26	**0.007**
Pachyvessel diameter change (*μ*m)	11.48 ± 30.55	15.43 ± 23.87	0.577
Choroidal cavern change	0.06 ± 0.73	0.03 ± 1.03	0.892

BCVA, best-corrected visual acuity; CMT, central macular thickness; CT, choroidal thickness.

p-values < 0.05 that are statistically significant are in bold font.

There were significant difference in BCVA, CMT, CT between two groups (p = 0.012, p = 0.005, p = 0.007, respectively).

### Factors affecting visual outcome after treatment in the CSC with CNV group

Univariate analysis showed that CT before treatment (B = 0.002; p = 0.026) and pachyvessel diameters before treatment (B = 0.002; p = 0.001) were significant factors affecting the final visual acuity in CSC with CNV group, which meant that thicker choroid and larger pachyvessel at baseline were significantly associated to poorer final visual acuity in CSC with CNV group (Table 4).

**Table 4:** Univariate and multivariate linear mixed models for associations between various clinical parameters and visual outcome after treatment in Group 1 (CSC with CNV).

	Univariate		Multivariate	
	B (95% CI)	p-value	B (95% CI)	p-value
Age	0.011 (0.000 to 0.021)	0.050	-	**-**
Sex	−0.024 (−0.310 to 0.262)	0.865	-	-
IOP	0.008 (−0.035 to 0.051)	0.711	-	-
SE	0.046 (−0.020 to 0.113)	0.165	-	-
CMT	0.001 (0.000 to 0.003)	0.142	-	-
CT	0.002 (0.000 to 0.004)	**0.026**	0.001 (−0.001 to 0.003)	0.247
Pachyvessel diameter	0.002 (0.001 to 0.004)	**0.001**	0.002 (0.001 to 0.004)	**0.001**
Choroidal caverns	-0.007 (-0.081 to 0.067)	0.847	-	-

CI, confidence interval; IOP, intraocular pressure; SE, spherical equivalent; CMT, central macular thickness; CT choroidal thickness

p-values < 0.05 that are statistically significant are in bold font.

In multivariate analysis, only baseline pachyvessel diameter (B = 0.002; p = 0.001) exhibited significant results.

## Discussion

Chronic CSC can lead to progressive visual dysfunction due to persistent serous retinal detachment, photoreceptor damage, and RPE atrophy [25]. It may be complicated with CNV known to be associated with decreased visual acuity [15]. Due to its poor prognosis, exact and prompt detection of CNV is imperative. Conventional imaging methods, such as FA and ICGA, cannot show NV apparently. The dye leakage in FA may prevent the effective NV visualization. Specifically in chronic CSC, sometimes even the clear leakage, pooling, and staining effect are not so obvious [21]. The use of OCTA allows for easier identification of NV in CSC. Quaranta-El Maftouhi et al. [18] reported that OCTA can be used to detect NV in CSC that could not be identified by FA or ICGA. Thus, the use of OCTA is invaluable for detecting CNV and developing a therapeutic plan to address chronic CSC.

In the CSC with CNV group (Group 1), CMT, CT, and pachyvessel diameter were significantly reduced after IVB. Visual acuity also improved significantly one month after treatment with IVB. Anti-VEGF treatment for chronic CSC with CNV is well-established [26]. Schworm et al. [27] reported that extended (6-month) anti-VEGF therapy yielded CMT reduction and improved visual outcome in chronic CSC with CNV. Similarily, Matsumoto et al. [28] reported that anti-VEGF therapy improved BCVA and CMT in pachychoroid neovasculopathy over a 2-year period. These results are consistent with those of our study. The SRF in CSC may originate from CNV activity and anti-VEGF treatment may be the most appropriate in cases showing exudation from the CNV [29]. After all, OCTA is an advanced diagnostic modality for CSC and if CNV was discovered by OCTA in chronic CSC patients, IVB could be proper treatment to achieve better visual and anatomical outcomes.

On OCT, CSC usually manifests as prominent and diffuse choroidal thickening with diffusely spread pachyvessels [30]. Increased CT is presumed to result mainly from the dilatation of pachyvessels (the choroidal vessels in Haller's layer) [31]. Baek et al. [30] reported that the presence of pachyvessels was closely correlated with a greater CT and higher choroidal vascular density. Dansingani et al. [32] showed that, in the case of pachychoroid spectrum diseases, the site of maximal CT in swept-source OCT choroidal thickness maps had dilated pachyvessels. In the CSC with CNV group in our study, CT and pachyvessel diameter were reduced after IVB. Anti-VEGF agents are known to affect choroidal circulation. It may reduce choroidal hyper-permeability by suppressing nitric oxide production, thereby reducing CT [33]. VEGF inhibition may also induce pachyvessel constriction, which would promote CT reduction.

Compared to Group1, the Group 2 (CSC without CNV) patients showed significantly larger CT diameters before treatment. Savastano et al. [21] reported a greater CT in cases of CSC without versus with CNV, similar to the results of our study. Moreira-Neto et al. reported atrophy of choriocapillaris in some areas surrounding CNV lesions in cases showing AMD [34]. In our study, sectoral choroidal atrophy caused by CNV would have resulted in thinner CT in Group 1. Further studies are needed to better understand this phenomenon. Regarding therapeutic results, there was no significant change in CMT or CT after IVB in Group 2. Moreover, visual acuity did not improve; only pachyvessel diameter was significantly reduced at 1 month after IVB treatment. This suggests that although anti-VEGF treatment reduced choroidal hyperpermeability, it did not significantly reduce CMT via SRF absorption, nor improve the visual outcome in Group 2. The use of IVB for treating chronic CSC has long been controversial, due to the large variation in therapeutic efficacy among previous studies [35,36]. In a meta-analysis, visual acuity and CMT at 6 months after IVB had not improved significantly [35]. Thus, they failed to demonstrate a positive effect of IVB on CSC. On the other hand, Chung et al. [36] reported that IVB treatment reduced CMT and improved visual outcomes in chronic and recurrent CSC over a 12-month period. Although the direct comparison of these studies is not appropriate because of different follow-up durations and the number of treatments (the number of IVB injections, 1.3 and 3.7, respectively), the effect of IVB in chronic CSC was contentious. However, these previous studies did not analyze the patients according to the presence or absence of CNV on OCTA. Thus, the results of these previous studies may be attributable to their CSC patients not being classified according to the presence or absence of CNV through OCTA. In our study, the CSC without CNV group showed a poorer response to IVB compared than the CSC with CNV group. This implies that, if CNV is not detected by OCTA in chronic CSC, other conventional treatments such as PDT and focal laser photocoagulation should be considered rather than IVB.

It is known that CSC with CNV is associated with a poorer visual outcome compared to CSC without CNV [15,16]. However, few studies have addressed the prognostic factors in chronic CSC with CNV. Thus, we attempted to identify factors affecting the short-term visual outcome after treatment for CSC with CNV group. In our study, a thinner choroid, and especially a smaller pachyvessel diameter at baseline, were correlated with better visual acuity. As mentioned above, because CT changes may be affected by pachyvessels, pachyvessel diameter may be a more important parameter with respect to short-term visual outcome; the larger the pachyvessels, the more likely that the choroid has been damaged. In the severely damaged choroid, the response to anti-VEGF may be poor, which may in turn result in a poor visual outcome. On the other hand, Kim et al. [37] demonstrated that a good short-term response to IVB in CSC could be expected with thicker choroid. However, they did not consider the presence of CNV revealed by OCTA, because their study was performed before OCTA had become widespread; thus it may have included patients with CNV showing a good response to IVB.

The limitations of our study included its retrospective nature and small sample size. Also, our study did not compare efficacy among different anti-VEGF drugs. Thus, further studies including large numbers of patients are needed to compare the long-term outcomes of various anti-VEGF drugs. The evaluation of changes in choroidal vessels after anti-VEGF treatment using OCTA such as quantitative analyses of choriocapillaris would be also meaningful in the future study.

In conclusion, IVB showed less effective short-term outcomes in chronic CSC patients without CNV than in patients with CNV, which was classified by OCTA. Therefore, other conventional treatments such as PDT or focal laser may be more appropriate for chronic CSC patients without CNV. Additionally, a thinner CT and smaller pachyvessels at baseline in patients with CNV were associated with better short-term visual outcomes after IVB, which demonstrated the importance of pachyvessels.

## Supporting information

S1 Data (XLSX)

## References

[1] Wang M, Munch IC, Hasler PW, Prunte C, Larsen M. Central serous chorioretinopathy. Acta Ophthalmology. 2008;86(2):126–45. 10.1111/j.1600-0420.2007.00889.x 17662099

[2] Yannuzzi LA. Central serous chorioretinopathy: A personal perspective. American journal of ophthalmology. 2010;149(3):361–3. 10.1016/j.ajo.2009.11.017 20172062

[3] Levine R, Brucker AJ and Robinson F. Long-term follow up of idiopathic central serous chorioretinopathy by fluorescein angiography. Ophthalmology. 1989;96(6):854–9. 10.1016/s0161-6420(89)32810-7 2740080

[4] Shiraki K, Moriwaki M, Matsumoto M, Yanagihara N, Yasunari T, Miki T. Long-term follow-up of severe central serous chorioretinopathy using indocyanine green angiography. International Ophthalmology. 1997;21(5):245–53. 10.1023/a:1006038621426 9756431

[5] Brancato R, Scialdone A, Pece A, Coscas G, Binaghi M. Eight-year follow up of central serous chorioretinopathy with and without laser treatment. Graefe's Archive for Clinical and Experimental Ophthalmology. 1987;225(3):166–8. 10.1007/BF02175443 3609756

[6] Arsan A, Kanar HS, Sonmez A. Visual outcomes and anatomic changes after sub-threshold micropulse yellow laser (577-nm) treatment for chronic central serous chorioretinopathy: long-term follow-up. Eye. 2018;32(4):726–33. 10.1038/eye.2017.293 29303148 PMC5898868

[7] Fung AT, Yannuzzi LA, Freund KB. Type 1 (sub-retinal pigment epithelial) neovascularization in central serous chorioretinopathy masquerading as neovascular age-related macular degeneration. Retina. 2012;32(9):1829–37. 10.1097/IAE.0b013e3182680a66 22850219

[8] Huang WC, Chen WL, Tsai YY, Chiang CC, Lin JM. Intravitreal bevacizumab for treatment of chronic central serous chorioretinopathy. Eye. 2009;23(2):488–9. 10.1038/eye.2008.55 18344956

[9] Inoue M, Kadonosono K, Watanabe Y, Kobayashi S, Yamane S, Arakawa A. Results of one-year follow-up examinations after intravitreal bevacizumab administration for chronic central serous chorioretinopathy. Ophthalmologica. 2011;225(1):37–40. 10.1159/000314709 20693820

[10] Schaal KB, Hoeh AE, Scheuerle A, Schuett F, Dithmar S. Intravitreal bevacizumab for treatment of chronic central serous chorioretinopathy. European Journal of Ophthalmology. 2009;19(4): 613–7. 10.1177/112067210901900415 19551677

[11] Chan WM, Lai TY, Liu DT, Lam DS. Intravitreal bevacizumab (avastin) for choroidal neovascularization secondary to central serous chorioretinopathy, secondary to punctate inner choroidopathy, or of idiopathic origin. American journal of ophthalmology. 2007;143(6):977–83. 10.1016/j.ajo.2007.02.039 17459318

[12] Coscas G, Lupidi M, Coscas F. Optical coherence tomography angiography in healthy subjects. Developments in Ophthalmology. 2016;56:37–44. 10.1159/000442775 27023473

[13] Spaide RF, Fujimoto JG, Waheed NK, Sadda SR, Staurenghi G. Optical coherence tomography angiography. Progress in Retinal and Eye Research. 2018;64(5):1–55. 29229445 10.1016/j.preteyeres.2017.11.003 PMC6404988

[14] Bonini Filho MA, de Carlo TE, Ferrara D, Adhi M, Baumal CR, Witkin AJ, et al Association of choroidal neovascularization and central serous chorioretinopathy with optical coherence tomography angiography. JAMA Ophthalmology. 2015;133(8):899–906. 10.1001/jamaophthalmol.2015.1320 25996386 PMC4721607

[15] Loo RH, Scott IU, Flynn HW Jr, Gass JD, Murray TG, Lewis ML, et al Factors associated with reduced visual acuity during long-term follow-up of patients with idiopathic central serous chorioretinopathy. Retina. 2002;22(1):19–24. 10.1097/00006982-200202000-00004 11884873

[16] Sulzbacher F, Schütze C, Burgmüller M, Vécsei-Marlovits PV, Weingessel B. Clinical evaluation of neovascular and non-neovascular chronic central serous chorioretinopathy (CSC) diagnosed by swept source optical coherence tomography angiography (SS OCTA) Graefe's Archive for Clinical and Experimental Ophthalmology. 2019; 257(8): 1581–90. 10.1007/s00417-019-04297-z 31037488

[17] Dansingani KK, Balaratnasingam C, Klufas MA, Sarraf D, Freund KB. Optical coherence tomography angiography of shallow irregular pigment epithelial detachments in pachychoroid spectrum disease. American journal of ophthalmology. 2015;160(6):1243–54. 10.1016/j.ajo.2015.08.028 26319161

[18] Quaranta-El Maftouhi M, El Maftouhi A, Eandi CM. Chronic central serous chorioretinopathy imaged by optical coherence tomographic angiography. American journal of ophthalmology. 2015;160(3):581–7. 10.1016/j.ajo.2015.06.016 26133250

[19] de Carlo TE, Rosenblatt A, Goldstein M, Baumal CR, Loewenstein A, Duker JS. Vascularization of irregular retinal pigment epithelial detachments in chronic central serous chorioretinopathy evaluated with OCT angiography. Ophthalmic Surgery, Lasers & Imaging Retina. 2016;47(2):128–133. 10.3928/23258160-20160126-05 26878445

[20] Bousquet E, Bonnin S, Mrejen S, Krivosic V, Tadayoni R, Gaudric A. Optical coherence tomography angiography of flat irregular pigment epithelium detachment in chronic central serous chorioretinopathy. Retina. 2018;38(3):629–38. 10.1097/IAE.0000000000001580 28267114

[21] Savastano MC, Rispoli M, Lumbroso B. The incidence of neovascularization in central serous chorioretinopathy by optical coherence tomography angiography. Retina. 2020;00(4):1–7. 10.1097/IAE.0000000000002810 32310626 PMC7819522

[22] Nagai N, Suzuki M, Minami S, Kurihara T, Kamoshita M, Sonobe H, et al Dynamic changes in choroidal conditions during anti-vascular endothelial growth factor therapy in polypoidal choroidal vasculopathy. Scientific Reports. 2019;6;9(1):11389 10.1038/s41598-019-47738-9 31388029 PMC6684594

[23] Matsumoto H, Mukai R, Kikuchi Y, Morimoto M, Akiyama H. One-year outcomes of half-fluence photodynamic therapy combined with intravitreal injection of aflibercept for pachychoroid neovasculopathy without polypoidal lesions. Japanese Journal of Ophthalmology. 2020;3;64(2):203–209. 10.1007/s10384-020-00722-7 32016666

[24] Lee MW, Park HJ, Shin YI, Lee WH, Lim HB, Kim JY. Comparison of choroidal thickness measurements using swept source and spectral domain optical coherence tomography in pachychoroid diseases. PLoS One. 2020;26;15(2):e0229134 10.1371/journal.pone.0229134 32101541 PMC7043756

[25] Nicholson B, Noble J, Forooghian F, Meyerle C. Central serous chorioretinopathy: update on pathophysiology and treatment. Survey of Ophthalmology. 2013;58(2):103–26. 10.1016/j.survophthal.2012.07.004 23410821 PMC3574296

[26] Montero JA Q., Ruiz-Moreno JM, Fernandez-Muñoz M. Intravitreal bevacizumab to treat choroidal neovascularization following photodynamic therapy in central serous choroidopathy. European Journal of Ophthalmology. 2011;21(4):503–5. 10.5301/EJO.2011.6290 21279982

[27] Schworm B, Luft N, Keidel LF, Hagenau F, Kern C, Herold T, et al Response of neovascular central serous chorioretinopathy to an extended upload of anti-VEGF agents. Graefe's Archive for Clinical and Experimental Ophthalmology. 2020;258(5):1013–21. 10.1007/s00417-020-04623-w 32112141

[28] Matsumoto H, Hiroe T, Morimoto M, Mimura K, Ito A, Akiyama H. Efficacy of treat‑and‑extend regimen with aflibercept for pachychoroid neovasculopathy and Type 1 neovascular age‑related macular degeneration. Japanese journal of Ophthalmology. 2018;62(2):144–50. 10.1007/s10384-018-0562-0 29411171

[29] Lai TYY, Staurenghi G, Lanzetta P, Holz FG, Liew SHM, Desset-Brethes S, et al Efficacy and safety of ranibizumab for the treatment of choroidal neovascularization due to uncommon cause: Twelve-Month results of the Minerva study. Retina. 2018;38(8):1464–77. 10.1097/IAE.0000000000001744 28704254 PMC6086222

[30] Baek J, Lee JH, Jung BJ, Kook L, Lee WK. Morphologic features of large choroidal vessel layer: age-related macular degeneration, polypoidal choroidal vasculopathy, and central serous chorioretinopathy. Graefe's Archive for Clinical and Experimental Ophthalmology. 2018;256(12):2309–17. 10.1007/s00417-018-4143-1 30259090

[31] Baek J, Lee JH, Chung BJ, Lee K, Lee WK. Choroidal morphology under pachydrusen. Clinical & experimental Ophthalmology. 2019;47(4):498–504. 10.1111/ceo.13438 30393991

[32] Dansingani KK, Balaratnasingam C, Naysan J, Freund KB. En face imaging of pachychoroid spectrum disorders with swept-source optical coherence tomography. Retina. 2016;36(3):499–516. 10.1097/IAE.0000000000000742 26335436

[33] Padrón‑Pérez N, Arias L, Rubio M, Lorenzo D, García‑Bru P, Català‑Mora J. Changes in choroidal thickness after intravitreal injection of anti‑vascular endothelial growth factorin pachychoroid neovasculopathy. Investigative ophthalmology & visual science. 2018;59(2):1119–24. 29490349 10.1167/iovs.17-22144

[34] Moreira-Neto CA, Moult EM, Fujimoto JG, Waheed NK, Ferrara D. Choriocapillaris Loss in Advanced Age-Related Macular Degeneration. Journal of Ophthalmology. 2018;30:8125267 10.1155/2018/8125267 29651346 PMC5831971

[35] Chung YR, Seo EJ, Lew HM, Lee KH. Lack of positive effect of intravitreal bevacizumab in central serous chorioretinopathy: meta-analysis and review. Eye. 2013;27(12):1339–46. 10.1038/eye.2013.236 24202051 PMC3869506

[36] Chung YR, Kim JW, Song JH, Park A, Kim MH. Twelve-month efficacy of intravitreal bevacizumab injection for chronic, atypical, or recurrent central serous chorioretinopathy. Retina. 2019;39(1):134–42. 10.1097/IAE.0000000000001917 29077604

[37] Kim GA, Rim TH, Lee SC, Byeon SH, Koh HJ, Kim SS, et al Clinical characteristics of responders to intravitreal bevacizumab in central serous chorioretinopathy patients. Eye. 2015;29(6):732–40. 10.1038/eye.2015.58 25952951 PMC4469674

